# Preliminary guideline- and pathophysiology-based protocols for neurocritical care

**DOI:** 10.1186/s40560-018-0316-6

**Published:** 2018-08-07

**Authors:** Yasuhiro Norisue, Yoshihisa Fujimoto, Kazuma Nakagawa

**Affiliations:** 1 Department of Emergency and Critical Care Medicine, Tokyo Bay Urayasu Ichikawa Medical Center, 3-4-32 Todaijima, Urayasu, Chiba 2790001 Japan; 2 Department of Emergency and Critical Care Medicine, St. Marianna University Hospital, 2-16-1, Sugao, Kawasaki, Kanagawa 2168511 Japan; 3 Department of Medicine, Division of Neurology, John A. Burns School of Medicine University of Hawai`i, 1301 Punchbowl Street, Honolulu, HI 96813 USA

**Keywords:** Neurocritical care, Protocols, Guidelines, Pathophysiology, Shivering, Neurological prognostication, Delayed cerebral ischemia, Nonconvulsive status epilepticus, Psychosis, Seizure

## Abstract

**Background:**

Because of the complex pathophysiological processes involved, neurocritical care has been driven by anecdotal experience and physician preferences, which has led to care variation worldwide. Standardization of practice has improved outcomes for many of the critical conditions encountered in the intensive care unit.

**Main body:**

In this review article, we introduce preliminary guideline- and pathophysiology-based protocols for (1) prompt shivering management, (2) traumatic brain injury and intracranial pressure management, (3) neurological prognostication after cardiac arrest, (4) delayed cerebral ischemia after subarachnoid hemorrhage, (5) nonconvulsive status epilepticus, and (6) acute or subacute psychosis and seizure.

**Conclusion:**

These tentative protocols may be useful tools for bedside clinicians who need to provide consistent, standardized care in a dynamic clinical environment. Because most of the contents of presented protocol are not supported by evidence, they should be validated in a prospective controlled study in future. We suggest that these protocols should be regarded as drafts to be tailored to the systems, environments, and clinician preferences in each institution.

## Background

The art of neurocritical care requires an understanding of the pathophysiology of the highly complex central nervous system. Because of its complexity and the lack of evidence, the approach to neurocritical care is often clinician-dependent, i.e., driven by anecdotal experience and physician preferences, which leads to care variation. Overall, standardization of practice has improved outcomes for many critical conditions in the intensive care unit; thus, greater emphasis should be placed on reducing variation in neurocritical care practice.

Guideline- and pathophysiology-based protocols are concise yet comprehensive and are useful for bedside clinicians who need to provide consistent, standardized practice in a dynamic clinical environment. We introduce five preliminary protocols in this article. Because most of the text of the protocols addresses management in neurocritical care fields that lack firm evidence, and because of the varied availability of medical resources among institutions, we recommend that these protocols be used as drafts to be customized for the systems, environments, and clinical preferences of each institution.

## Protocols

### Prompt shivering management (Fig. 1)

**Fig. 1:**
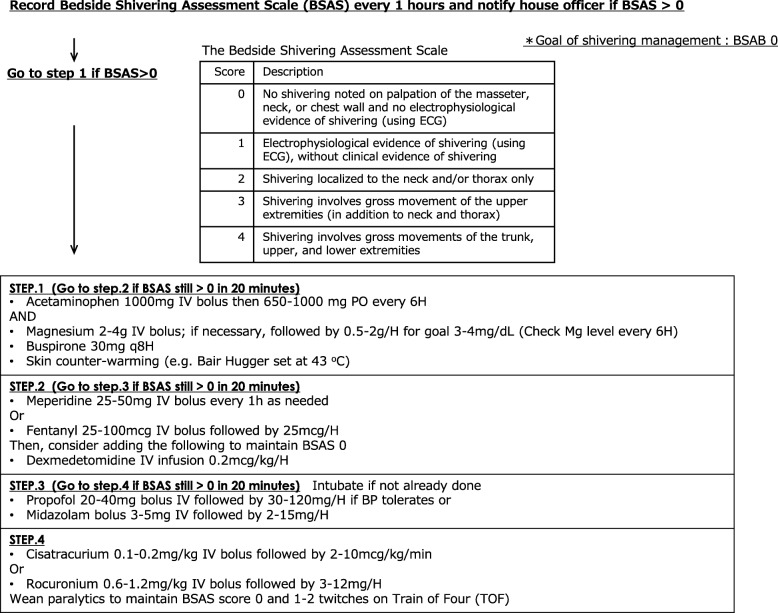
Preliminary protocol for prompt shivering management [1, 2]. Modified from Brophy [2] with permission. Abbreviations: BSAS Bedside Shivering Assessment Scale, ECG electrocardiogram, IV intravenously, PO per oral, H hour, min minute

Shivering is a physiological homeostatic response to maintain or raise temperature in hypothermia or fever when the set point temperature is elevated. However, shivering counteracts the effort of fever management and targeted temperature management (TTM)/therapeutic hypothermia, which are critical interventions to mitigate secondary brain injury. With inadequate shivering management, target temperature is difficult to achieve in a timely manner and may potentially worsen outcome. Furthermore, shivering increases the cerebral metabolic rate and may result in increased intracranial pressure (ICP) and brain oxygen consumption [1, 2]. Lastly, shivering increases the total body metabolic rate and total CO_2_ production, which may raise the partial pressure of CO_2_ and raise ICP. Therefore, shivering should be regarded as a neurological emergency requiring immediate control in patients with acute brain injury, and any protocol for shivering management should encourage clinicians to expedite treatment. The present draft proposal for shivering management refers to the Bedside Shivering Assessment Scale and shivering protocol proposed by Badjatia and Brophy, respectively, [1, 2] and was refined, based on our practice, to achieve prompt shivering control (Fig. 1).

### Traumatic brain injury and ICP management (Fig. 2)

**Fig. 2:**
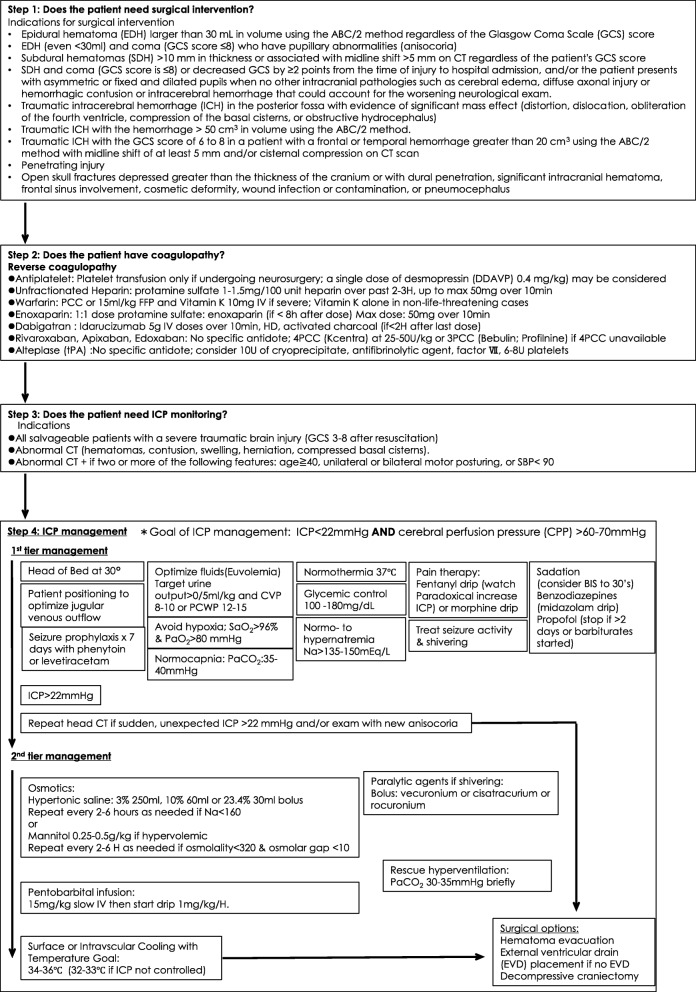
Preliminary protocol for traumatic brain injury and ICP management [4–8]. Abbreviations: CT computed tomography, SBP systolic blood pressure, U units, 3PCC 3-factor prothrombin complex concentrate, 4PCC 4-factor prothrombin complex concentrate, ICP intracranial pressure, CVP central venous pressure, PCWP pulmonary capillary wedge pressure, SaO_2_ arterial oxygen saturation, PaO_2_ arterial pressure of oxygen, PaCO_2_ arterial pressure of carbon dioxide

Intracranial hypertension, commonly defined as persistent elevation of ICP above 20–22 mmHg, is a relatively common neurologic complication seen after traumatic brain injury (TBI). If untreated, it can lead to cerebral ischemia, brain herniation, and possibly brain death. Therefore, care providers must promptly recognize the early clinical and radiographic features of elevated ICP and aggressively treat with a goal of reducing mortality and morbidity. The adult brain is a nearly incompressible substance enclosed in a fixed cranium. Therefore, ICP will inevitably be affected by a volume change in any of the three main intracranial components—cerebrospinal fluid (CSF), brain parenchyma, and blood [3]. In addition, if there is a new space-occupying lesion within the fixed cranium (i.e., hematoma), it will inevitably increase ICP. When assessing a patient with elevated ICP, it is important to determine whether the contributing factor is a focal, global, or mixed process since the treatment strategy may be different for each type of mass effect. If there is a focal, new mass-occupying lesion that is causing a regional mass effect and brain tissue compression, the first step is to consider surgical evacuation. Once the focal mass effect is ruled out or treated, global elevation of ICP must be addressed. The overarching strategy to control globally elevated ICP is to (1) optimize cerebral perfusion, oxygenation, and venous drainage; (2) prevent fever, hypercapnia, hyponatremia, hypo/hyperglycemia, and seizure; (3) provide adequate cerebral metabolic suppression with sedation; and (4) reduce cerebral edema with osmotic therapy. For refractory intracranial hypertension, treatments to be considered include pentobarbital-induced coma, therapeutic hypothermia, ventriculostomy placement for CSF diversion, and decompressive craniectomy. Ideally, a protocol for TBI management should include not only ICP control but also indications of initial surgical intervention for intracranial hematoma and basic management to prevent secondary brain injury. Step 1 (indications for surgical intervention) of the preliminary protocol is based on the recommendations by Bullock et al. [4–7], and step 2 (indications for ICP monitoring) and step 3 (basic management of TBI and ICP control) were developed in accordance with the guidelines for management of severe traumatic brain injury of the Brain Trauma Foundation and our practice (Fig. 2) [8].

### Neurological prognostication after cardiac arrest (Fig. 3)

**Fig. 3:**
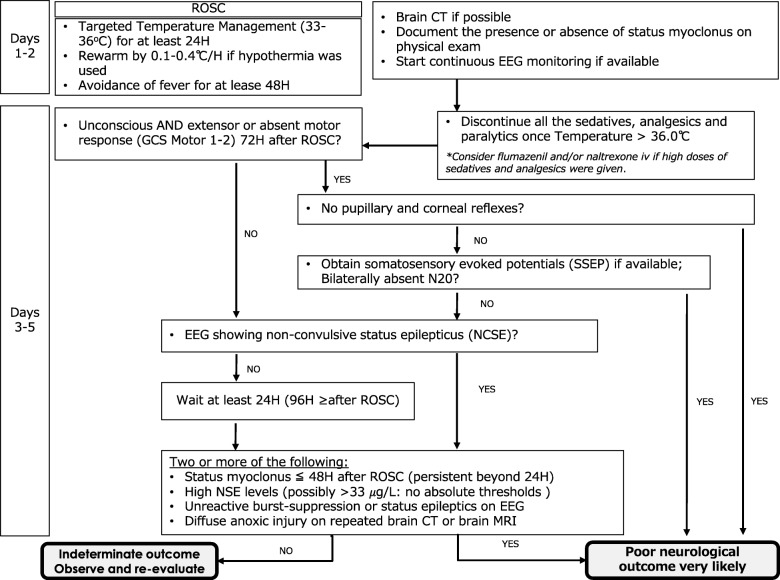
Preliminary protocol for neurological prognostication after cardiac arrest [13, 16]. Modified from Nolan [13] with permission. Abbreviations: ROSC return of spontaneous circulation, EEG electroencephalography

Cardiac arrest causes complete cessation of cerebral perfusion and rapidly depletes oxygen and glucose delivery to cerebral tissue. Cell death, which includes ion channel dysfunction and cell membrane destabilization, release of destructive enzymes, cell swelling, and, eventually, apoptosis, can begin within 5 min after complete cessation of cerebral perfusion [9–11]. After return of spontaneous circulation, neurological prognostication is essential because it enables clinicians to provide information to family members or surrogates who must consider decisions to limit care for patients with little hope of meaningful neurological recovery [11]. To date, there is no definitive diagnostic test to accurately predict functional outcome after cardiac arrest. Moreover, clinical findings shortly after cardiac arrest have little relationship to patient outcomes [12]. However, the use of a systematic approach allows for reliable prediction of a very poor neurological outcome (persistent vegetative state) and provides family members and surrogates with the information necessary to make decisions [13–16]. A protocol that clearly addresses “what to do next” would be helpful for clinicians. The present preliminary protocol is based on the 2015 European Society of Intensive Care Medicine Guidelines for Post-resuscitation Care [13], but includes additional detailed stepwise instructions (Fig. 3).

### Delayed cerebral ischemia after subarachnoid hemorrhage (Fig. 4)

**Fig. 4:**
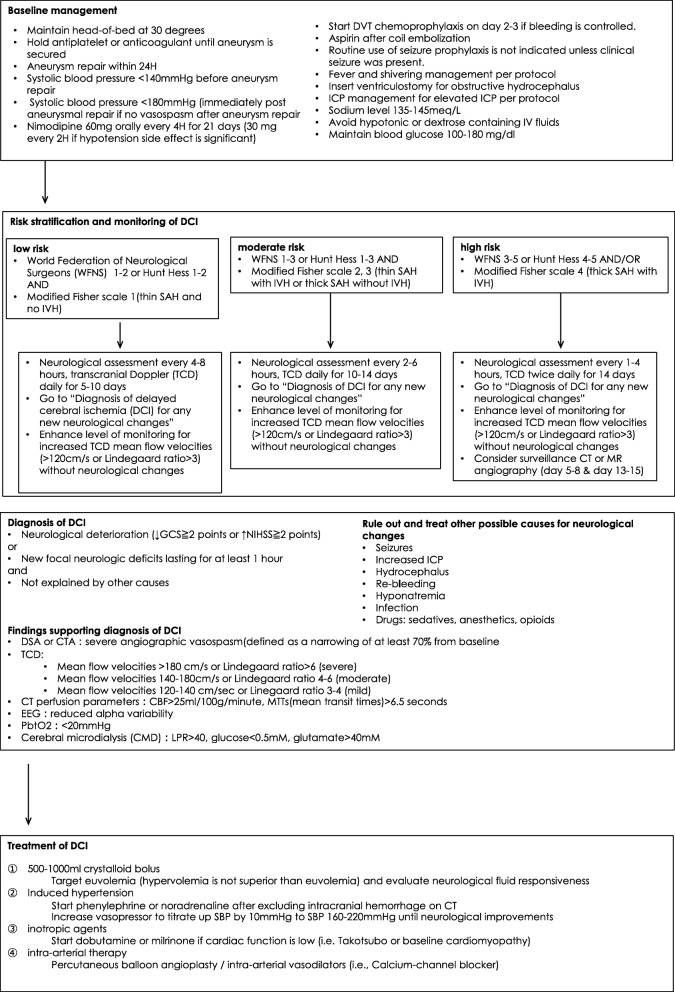
Preliminary protocol for monitoring and diagnosis of delayed cerebral ischemia after subarachnoid hemorrhage [23, 24]. Abbreviations: SAH subarachnoid hemorrhage, DVT deep vein thrombosis, IVH intraventricular hemorrhage

After subarachnoid hemorrhage, notably from a ruptured cerebral aneurysm, cerebral vasospasm can develop, thus leading to delayed cerebral ischemia (DCI) and possibly infarction. The mechanism of DCI is complex and is not solely attributed to large-vessel narrowing and associated low blood flow distally [17, 18]. Other postulated mechanisms are early brain injury, microcirculatory dysfunction with loss of cerebral autoregulation, cortical spreading depolarization, and microthrombosis [19, 20]. A DCI diagnosis is made clinically on the basis of symptoms such as new changes in mental status and neurologic deficits. Additional relevant information includes findings of CT or MRI angiography, digital subtraction angiography, and transcranial Doppler (TCD) ultrasound. In addition, other reversible causes of neurological changes must be ruled out, such as delayed hydrocephalus, nonconvulsive seizure, rebleeding, toxic-metabolic encephalopathy from infection, and medication side effects. To date, nimodipine, a calcium-channel blocker with cerebral vasodilatory effect, is the only drug that has been shown to improve neurological outcomes in patients with subarachnoid hemorrhage [21]. Other calcium-channel blockers, such as nicardipine, have been used in countries where nimodipine is unavailable [22] but have not been shown to improve outcomes. Hemodynamic augmentation to increase oxygen delivery to the brain, including volume optimization and induced hypertension, is the mainstay for management of new-onset DCI. For refractory cases where medical management is ineffective, intra-arterial interventions such as balloon angioplasty and intra-arterial administration of calcium-channel blockers are a second-line treatment [23]. A protocol should include risk stratification and stepwise treatment for DCI in individual patients. The present preliminary protocol describes basic management of subarachnoid hemorrhage and risk stratification and monitoring, based on our practice and the existing literature [24–26]. Diagnosis and management of DCI are based on recommendations from the neurocritical care society and our practice [23] (Fig. 4).

### Diagnosis of nonconvulsive status epilepticus (Fig. 5)

**Fig. 5:**
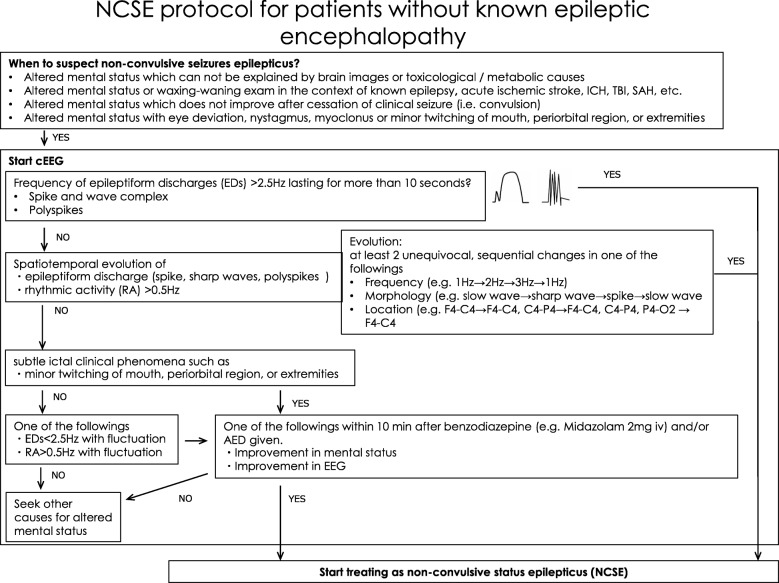
Preliminary protocol for diagnosis of nonconvulsive status epilepticus [32]. Abbreviations: AED anti-epileptic drug

Nonconvulsive status epilepticus (NCSE) is characterized by electrographic seizure activity without clinical convulsions in patients who do not fully recover consciousness between attacks [27]. Although the impact of treating NCSE on clinical outcomes has not been investigated in a randomized controlled trial, the prognosis of NCSE is believed to be poor if not treated since untreated seizure is associated with secondary brain injury [28–30]. A diagnosis of NCSE should be considered in any patient with discrepancies between his/her neurological findings and clinical history or imaging findings such as CT or MRI. The typical example is a patient who develops sudden unexpected neurological deterioration after successful management of a structural brain injury without new findings on CT or MRI. Although the condition is referred to as nonconvulsive, patients with NCSE may have subtle motor symptoms such as sustained eye deviation, nystagmus, lip smacking, and twitching in the face or extremities [31]. Definitive diagnosis requires electroencephalography (EEG), and continuous EEG monitoring increases the sensitivity and specificity of NCSE diagnosis. We attempted to develop an algorithmic protocol to interpret EEG and diagnose NCSE for bedside clinical use. Based on the current guidelines [27, 32], this draft protocol for diagnosis of NCSE is designed to simplify NCSE diagnosis and management (Fig. 5).

### Acute or subacute psychosis and seizure (Fig. 6)

**Fig. 6:**
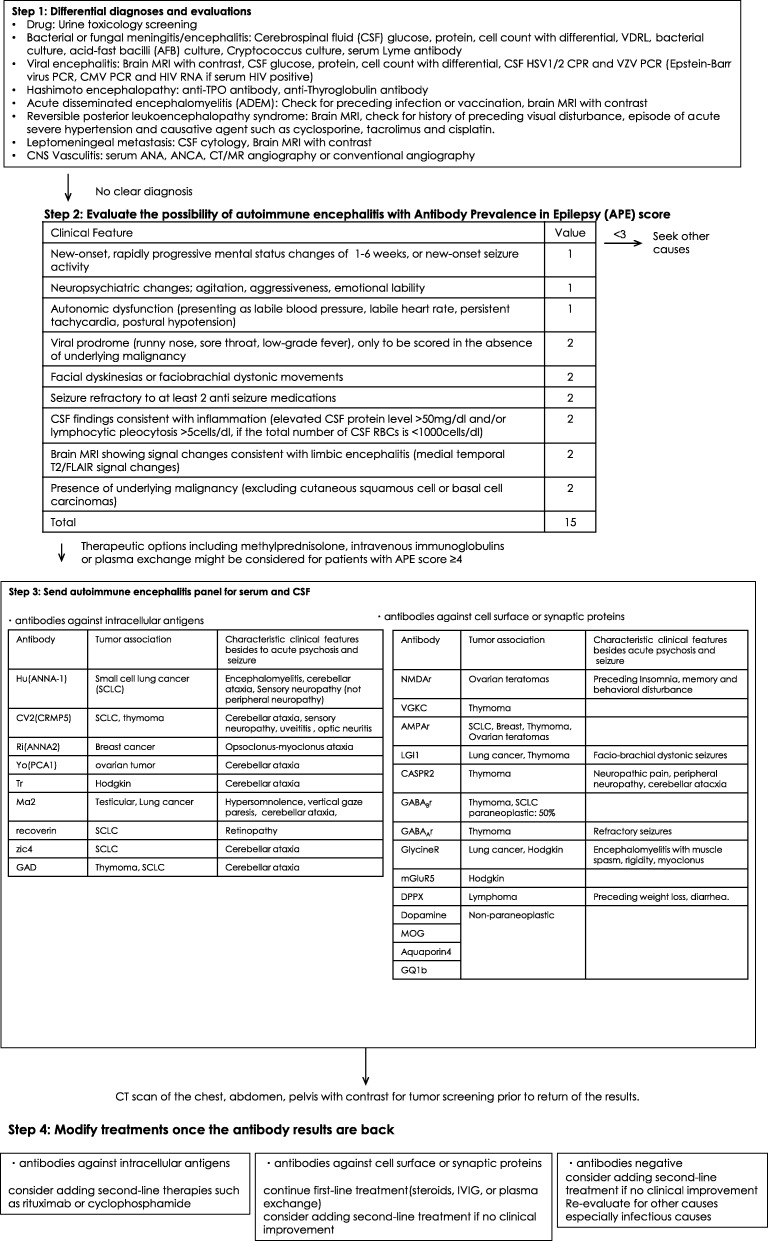
Preliminary protocol for acute or subacute psychosis and newly onset seizure [33, 35]. Modified from Dubey [33] with permission. Abbreviations: VDRL venereal disease research laboratory, HSV herpes simplex virus, VZV varicella zoster virus, HIV human immunodeficiency virus, CMV cytomegalovirus, Anti-TPO anti-thyroid peroxidase, ANA anti-nuclear antibody, ANCA antineutrophil cytoplasmic antibody

Many medical conditions and pharmacologic side effects can cause unexpected psychosis or seizure. However, some treatable and reversible conditions, such as viral encephalitis and autoimmune encephalitis, are frequently missed. Delays in diagnosis and treatment of encephalitis may result in poor neurological outcomes. It is essential to review all possible causes of unexpected psychosis and seizure and to start empirical treatment for a potentially treatable condition before obtaining all examination results. The present draft protocol for diagnosis and management of autoimmune encephalitis includes comprehensive differential diagnoses and algorithms for diagnostic evaluation and was developed on the basis of a comprehensive literature review by Francesc et al., antibody prevalence in epilepsy (APE) score [33], and empirical treatment for autoimmune encephalitis in cases of unexpected psychosis or seizure, as reflected by the expert opinions of the European Federation of the Neurological Societies (EFNS) task force [34] and our practice (Fig. 6).

## Conclusion

The present guideline- and pathophysiology-based protocols that can be customized for particular clinical environments may help providing consistent, standardized care in neurocritical care. Because most of the contents of presented protocol are not supported by evidence, they should be validated in a prospective controlled study in future.

## Abbreviations

CSF: Cerebrospinal fluid
DCI: Delayed cerebral ischemia
EEG: Electroencephalography
ICP: Intracranial pressure
NCSE: Nonconvulsive status epilepticus
TBI: Traumatic brain injury
TCD: Transcranial Doppler
TTM: Targeted temperature management
